# The effect of Traditional Chinese Medicine Tuina massage combined with family-based prevention on visual acuity in children and adolescents with myopia: A case report of single–group interventional study

**DOI:** 10.1097/MD.0000000000046414

**Published:** 2026-01-02

**Authors:** Jun Wu, Peng Wang, Shuqi Jia, Feng Ding, Caiyun Wang, Qianmin Su, Jihan Huang, Jing Wang

**Affiliations:** a Department of Physical Education, Shanghai University of Engineering Science, Shanghai, China; b School of Physical Education, Shanghai University of Sport, Shanghai, China; c Center for Clinical Drug Research, Shanghai University of Traditional Chinese Medicine, Shanghai, China; d School of Sports and Health, Shanghai Lixin University of Accounting and Finance, Shanghai, China.

**Keywords:** amblyopia, blue-light-blocking glasses, lutein, pediatric myopia, spinal manipulation, Traditional Chinese Medicine Tuina massage

## Abstract

**Objective:**

To evaluate the efficacy of Traditional Chinese Medicine Tuina massage combined with family-based preventive measures in improving visual acuity among children and adolescents with myopia.

**Methods:**

A single-arm intervention study enrolled 139 myopic participants aged 5 to 15 years. Over 12 weeks, participants received weekly Traditional Chinese Medicine Tuina massage sessions (3 times/week), including organ-system regulation, acupoint stimulation, and gentle spinal manipulation. Family-based interventions included blue-light-blocking glasses and lutein supplementation. Pre- and post-intervention visual acuity (binocular, better-eye, and worse-eye) were analyzed.

**Results:**

Post-intervention, all participants showed statistically significant improvements in visual acuity. Non-amblyopic patients exhibited greater improvement than amblyopic counterparts. The subgroup using blue-light-blocking glasses demonstrated the most pronounced gains, whereas lutein supplementation alone did not reach statistical significance.

**Conclusion:**

Traditional Chinese Medicine Tuina massage, particularly when combined with blue-light-blocking glasses, effectively improves visual acuity in pediatric myopia, with enhanced effects in non-amblyopic cases. Family-based measures are a critical adjunct, though lutein’s role requires further investigation.

## 1. Introduction

Myopia has emerged as a significant global public health concern.^[1]^ According to the World Health Organization, it is projected that by 2050, around 49.8% of the world’s population will be affected by this condition.^[2]^ The prevalence of myopia in China has been rising steadily, positioning it as the country with the highest number of myopic individuals worldwide.^[3]^ Individuals with myopia not only face substantial challenges in their daily lives but are also at a significantly increased risk of developing conditions such as glaucoma,^[4]^ cataracts,^[5]^ macular degeneration,^[6]^ and retinal detachment,^[7]^ in comparison to the general population. The 2023 national screening results for myopia among children and adolescents revealed a 60.4% detection rate for visual impairment, with a 51.0% detection rate specifically for myopia. The detection rates were 20.2% for grades 1 to 3 in primary school, 46.8% for grades 4 to 6, 67.3% for middle school, and 80.5% for high school students.^[8]^ The prevention and management of myopia in children and adolescents requires urgent attention and prompt action.

Both Traditional Chinese Medicine and Western medicine highlight the importance of enhancing blood flow to the vertebral arteries to improve vision. According to Traditional Chinese Medicine, Qi is the fundamental life force that sustains health.^[9]^ The organs serve as the primary source of Qi, which flows towards the eyes, nourishing them with blood to support vision.^[10]^ The meridians act as channels through which Qi and blood circulate, enabling the organs to generate Qi and blood, which are subsequently delivered to the eyes, thereby nourishing the eyeballs.^[11]^ Therefore, efficient circulation and sufficient blood supply are essential for maintaining vision. The primary blood supply to the eyes is derived from the ophthalmic and vertebral arteries. Although the ophthalmic artery is less vulnerable to compression by the vertebral bodies in the cervical region, inadequate blood flow from the vertebral artery can compromise the overall perfusion of the internal carotid artery, indirectly resulting in ocular ischemia and visual impairment.^[12]^ Studies in Western medicine have also demonstrated a significant correlation between blood flow in the vertebral artery and visual function. Tension in the suboccipital muscles may compress the vertebral artery or stimulate the sympathetic nerves, resulting in visual impairment.^[13]^ Relaxation of the suboccipital muscles, along with cervical spine adjustments, can significantly alleviate symptoms of blurred vision in patients.^[14]^ Dynamic angiography further confirms that cervical joint disorders, such as atlantoaxial dislocation, can lead to mechanical stenosis and compression of the vertebral artery, resulting in decreased blood flow.^[15]^

Currently, vision correction methods predominantly rely on Western medicine, utilizing external interventions such as corrective eyeglasses, orthokeratology lenses, and atropine eye drops. These methods are simple and effective, yet they may lead to side effects, including photophobia, dryness, eye inflammation, and in some cases, necessitate lifelong use of corrective lenses. In contrast, Traditional Chinese Medicine mainly utilizes acupuncture^[16,17]^ and Traditional Chinese Medicine Tuina massage (Chinese therapeutic massage)^[18,19]^ to improve vision. Numerous studies have demonstrated that both treatments can enhance ocular blood circulation, nourish the eyeballs, and dredge the meridians, thereby promoting the improvement of symptoms and the recovery of vision. Tuina massage, as a noninvasive therapy, is more readily accepted by patients. Most studies have concentrated on head and facial massage, as well as massage of the shoulders, neck, and back, all of which enhance vertebral artery blood flow, improve Qi and blood circulation to the eyes, and effectively relieve visual fatigue.^[11,18–20]^ Other studies have explored acupressure on the arms, demonstrating its effectiveness in improving the regulation of the pupillary sphincter and ciliary muscles, promoting retinal blood circulation, and yielding positive therapeutic results.^[21]^

These previous studies suggest that the issue of myopia in children and adolescents requires urgent attention. Traditional Chinese Medicine Tuina massage, as an effective method with minimal side effects, warrants further exploration. Tuina massage, as an effective treatment with minimal side effects, warrants further investigation. Moreover, current research seldom addresses familial preventive factors. Factors such as diet, visual habits, and nutritional supplementation in myopic children and adolescents may modulate treatment efficacy, yet the degree of their impact remains insufficiently explored. The integration of Traditional Chinese Medicine Tuina massage with family interventions strategies, as well as the role of family interventions in this context, necessitates further investigation. This exploratory clinical study investigates the impact of integrated Traditional Chinese Medicine Tuina massage and home-based myopia control strategies on visual acuity in pediatric populations through a single-arm trial design.

## 2. Methods

### 2.1. Study participants

This study employed a single-group trial design based on consecutive case recruitment. Since this is an exploratory study aimed at observing real-world clinical outcomes, no formal sample size estimation was conducted. Participants were recruited using consecutive sampling from patients at an ophthalmology clinic in Minhang District, Shanghai, between October 2019 and November 2021.

Diagnostic criteria: Diagnosis was based on the “Diagnostic Criteria and Efficacy Standards for Traditional Chinese Medicine Internal Medicine Diseases” (ZY/T001.1-94)^[22]^ of the People’s Republic of China. Mild myopia was defined as a spherical refractive error of −0.50 D to −3.00 D, moderate myopia as −3.00 D to −6.00 D, and high myopia as greater than −6.00 D. According to the “Chinese Expert Consensus on Amblyopia Prevention and Treatment (2021)”^[23]^ issued by the Strabismus and Pediatric Ophthalmology Group of the Chinese Ophthalmological Society, amblyopia is diagnosed if the best-corrected visual acuity in one or both eyes falls below the lower normal limit for the corresponding age group during the visual development period (the lower normal limit is 0.5 for children aged 3–5 years and 0.7 for children aged 6 years and above), with no other organic ocular pathologies present.

Inclusion criteria: Participants meeting the diagnostic criteria for myopia; Children and adolescents aged 5 to 18 years with myopia; No other vision intervention has been received before; and Willingness to undergo treatment and comply with medical instructions, with both the participant and their guardian being fully informed of the treatment process and providing written informed consent.

Exclusion criteria: Severe diseases such as cardiovascular, cerebrovascular, hepatic, renal, or hematological disorders; psychiatric conditions; skin injuries or dermatological conditions; Skeletal abnormalities, tuberculosis, tumors, fractures, or diseases of the cranial base and spinal canal; history of cervical spine surgery or congenital cervical spine deformities; Pathological myopia with ocular complications; and Long-term use of high-concentration atropine.

Criteria for exclusion, dropout, and trial termination: Participants who failed to complete treatment as prescribed, whose efficacy could not be evaluated due to incomplete data or other factors affecting the outcome; Worsening of the participant’s condition requiring a change in treatment approach; Adverse events during the intervention that necessitate discontinuation of the study; and Request by the participant or guardian to discontinue the study, or termination due to other reasons.

**Figure 1.**
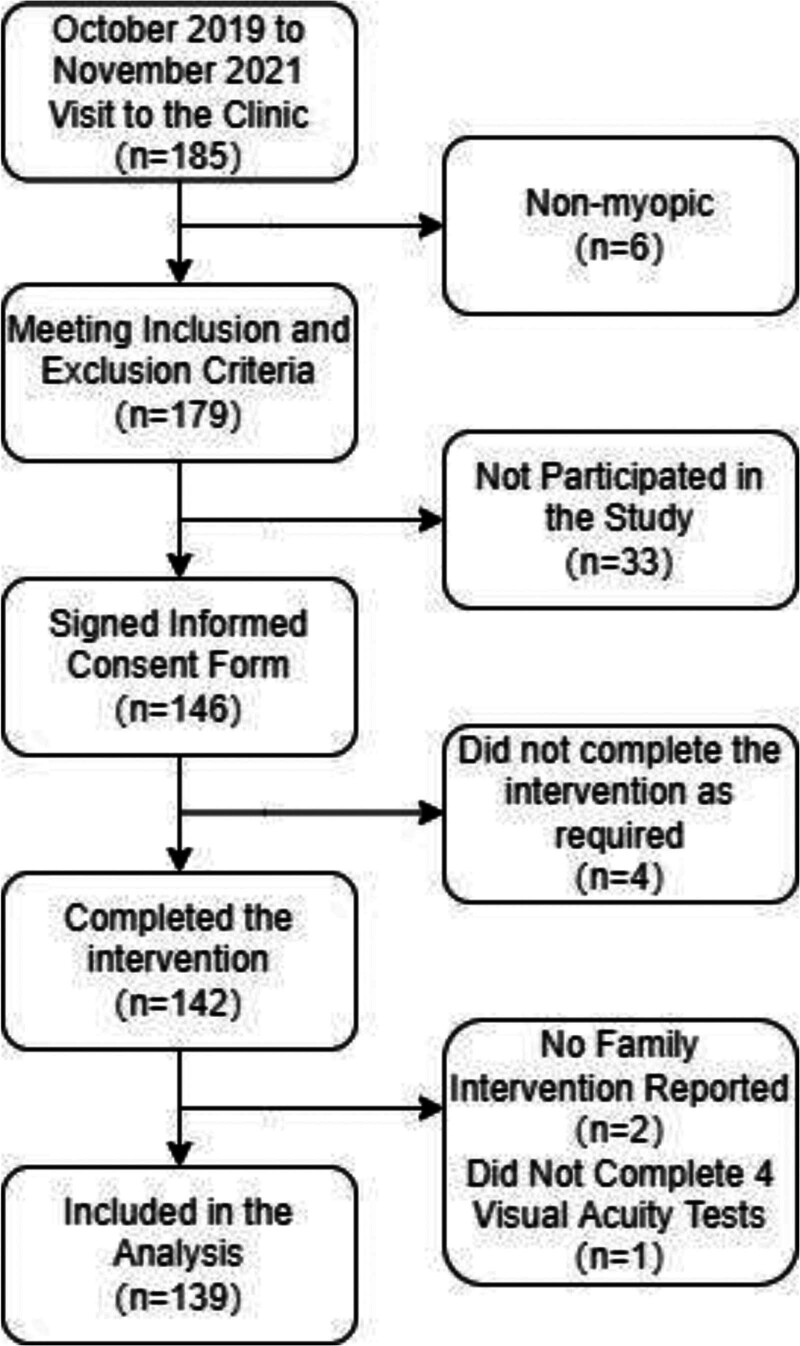
Participant inclusion process.

A total of 139 myopic children and adolescents were enrolled in this study, comprising 74 males and 65 females, aged 5 to 15 years, with a mean age of 9.64 years. The inclusion and exclusion process is depicted in Figure 1. All participants were informed of the study procedures, and written informed consent was obtained from their guardians. This study was conducted in accordance with the principles of the Declaration of Helsinki and was approved by Ethics Committee of the Shanghai University of Sport (102772023RT192).

### 2.2. Study design

This study employed a within-group multifactorial experimental design. The independent variables included time stages (0 week, 4 weeks, 8 weeks, 12 weeks), use of blue light-blocking glasses (yes/no), and use of lutein (yes/no), while the dependent variables included binocular vision, vision in the better eye, and vision in the poorer eye.

**Figure 2.**
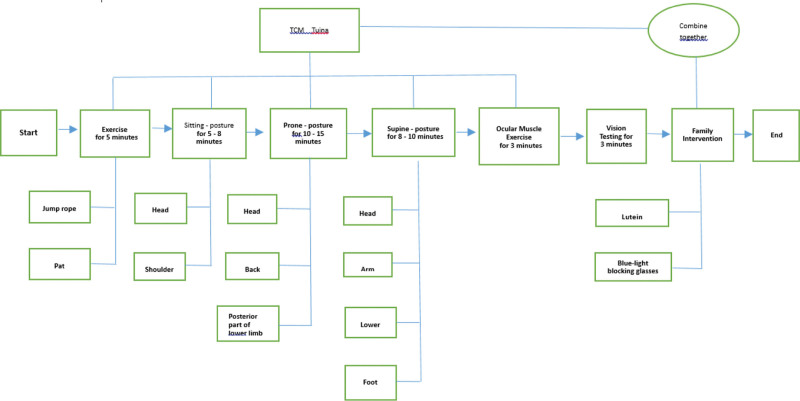
Visual health adjustment flowchart.

The intervention lasted for 12 weeks, with 2 to 3 sessions of Traditional Chinese Medicine Tuina massage per week, each lasting approximately 30 to 40 minutes. In addition, participants were instructed to follow family interventions measures as per the doctor’s recommendations. Compliance with these measures was assessed and recorded after each session. Vision was recorded at the end of each intervention session, with outcomes measured at the following time points: baseline (0 week), after 4 weeks of intervention, after 8 weeks of intervention, and after 12 weeks of intervention. The detailed procedure is shown in Figure 2.

#### 2.2.1. Traditional Chinese Medicine Tuina massage

The Traditional Chinese Medicine Tuina massage session lasts about 40 minutes, and the specific steps are as follows:

First, there is a 5-minute warm-up. It includes 2 minutes of medium-speed skipping rope (180–200 jumps, divided into 1–2 groups, aiming to stimulate the circulation of Qi and blood), and 3 minutes of tapping therapy (patting exercise). Pat the Zhongfu (LU1) and Yunmen (LU2) acupoints with the palm, and pat the Pishu (BL20), Weishu (BL21), Shenshu (BL23) and Mingmen (GV4) acupoints with the back of the hand to dredge the meridians and regulate the functions of the viscera.

Then, an 8-minute sitting massage follows. Apply finger-pressure on acupoints on the head and face such as Yintang (EX-HN3), Cuanzhu (BL2), Yuyao (EX-HN4), Sizhukong (TE23), Taiyang (EX-HN5), Yangbai (GB14), Tongziliao (GB1), Chengqi (ST1), Sibai (ST2), Juliao (ST3), Quanliao (SI18), Yingxiang (LI20), and acupoints on the neck and shoulders like Dazhui (GV14), Fengchi (GB20) (30 seconds for each acupoint). The purpose is to dredge the meridians connecting the head, eyes and viscera, and enhance the flow of Qi and blood to nourish the eyes.

Next is a 15-minute prone massage. First, use 4 fingers to knead and press acupoints such as Fengchi (GB20), Fengfu (GV16) and Yifeng (TE17) on the occipital region for 2 to 3 minutes to relax the neck muscles and dredge the blood flow from the carotid and vertebral arteries to the head. Then, perform a 1-minute gentle upward grasping of the neck to relax the sympathetic nerves. Finally, use the thumb to apply finger-pressure on acupoints of the Bladder Meridian such as Feishu (BL13), Xinshu (BL15), Ganshu (BL18), Danshu (BL19), Pishu (BL20), Weishu (BL21), Shenshu (BL23), Weizhong (BL40) and Chengshan (BL57) for 11 minutes to dredge the meridians between the viscera and the head and promote the upward flow of Yang Qi to the head.

After that, an 8-minute supine massage is carried out. Apply finger-pressure on acupoints of the leg meridians (30 seconds for each acupoint), including Xuehai (SP10), Yinlingquan (SP9), Sanyinjiao (SP6), Taibai (SP3) of the Spleen Meridian, Taichong (LR3) of the Liver Meridian, Taixi (KI3), Yongquan (KI1) of the Kidney Meridian (KI), Zusanli (ST36), Yanglingquan (GB34), Fenglong (ST40) of the Stomach Meridian, Neiguan (PC6), Laogong (PC8) of the Pericardium Meridian, and Waiguan (TE5) of the Sanjiao Meridian.

Subsequently, there is a 3-minute eye-muscle training. Place the hand 40 to 50 centimeters above the patient’s eyes and slowly perform up–down, left–right, 45° diagonal, and internal–external rotation movements (3 times each) to strengthen the extraocular muscles and ciliary muscles.

Finally, if there are problems such as spinal misalignment, pelvic tilt, leg-length difference or uneven shoulders, perform gentle osteopathic correction to ensure the correct position of organs, smooth meridians, and the supply of Qi and blood to the head and eyes. These problems are common in myopia/amblyopia patients due to bad postures (such as holding the pen too low and sitting for a long time). The key correction methods include kneading the iliopsoas muscle with the palm to correct the pelvis and applying acupoint-pressure on the levator scapulae muscle to restore the blood flow in the neck (vertebral artery/carotid artery) and prevent eye ischemia.

#### 2.2.2. Family interventions

Family interventions encompass several factors, with medical advice emphasizing dietary adjustments, such as avoiding cold or excessively sweet foods and beverages (e.g., ice cream, iced water, chocolate, and candy), drinking more hot water and eye-health-promoting teas (e.g., goji berry tea, red dates, chrysanthemum, and cassia seeds), and engaging in outdoor physical activities. However, these variables are difficult for researchers to control. Considering the practicality of these factors and their importance based on clinical experience, we selected 2 key family intervention factors: Blue light-blocking glasses: Participants are advised to wear appropriate glasses to reduce blue light exposure while using electronic devices. If the glasses are worn for the majority of screen time, this is classified as “Yes”; if the wear time is less than half of the screen time, it is classified as “No”; Lutein supplementation: Children under 8 years are prescribed 4 to 6 mg daily, while those 9 years and older receive 6 to 8 mg daily. If the child meets the recommended dosage for 6 or 7 days per week, it is classified as “Yes”; otherwise, it is classified as “No.”

### 2.3. Measures

Demographic Information: gender, age, height, history of chronic rhinitis (referred to as “rhinitis”), and whether at least one parent has myopia of 600 degrees or more (referred to as “parental myopia”); Visual Acuity: assessment of both eyes, better eye, and worse eye at different time points (0 week, 4 weeks, 8 weeks, 12 weeks); and Family Intervention Factors: whether the participant adhered to the prescribed use of blue light-blocking glasses (“blue light protection”) and lutein supplementation (“lutein”).

### 2.4. Data analysis

This study adopted a single–group trial design. To ensure the accuracy of the analysis results, the per-protocol set analysis method was used, and only the data of subjects who strictly adhered to the study protocol and completed all scheduled treatments or tasks were included. SPSS 29.0 (IBM Corporation, Chicago) was used for statistical analysis. Due to the limited sample size, moderate and high myopia cases were combined for analysis. The key methods included: categorical data were described as n (%), and the χ^2^ test was used for comparison; for visual acuity data (approximately normally distributed), paired *t*-tests were used for within-group comparisons (comparisons between baseline and week 4/8/12), and Cohen *d* effect size (95% confidence interval (CI)) was calculated, and line charts were used for trend visualization; one - way analysis of variance (ANOVA) (effect size: η^2^) was used for continuous data, and if there were significant differences, Bonferroni post-hoc tests were performed; repeated-measures ANOVA was used to analyze the factors affecting visual acuity (time, myopia degree, amblyopia, lutein use, blue-light glasses), and the Greenhouse–Geisser correction was used to adjust for non-sphericity; all tests were two-sided, and the significance level α = 0.05. The effect size calculation formulas included Cohen *d* (mean difference/standard deviation of the difference) and Cramér *V* for the χ^2^ test.

## 3. Results

### 3.1. Demographic information of participants

The demographic information of the participants is shown in Table 1. Among the participants, 9 had moderate-to-severe myopia with amblyopia, 64 had moderate-to-severe myopia without amblyopia, 8 had mild myopia with amblyopia, and 58 had mild myopia without amblyopia. There were no significant differences between groups in terms of gender ratio, age, height, or chronic rhinitis. However, a significant difference was observed in the parental myopia status (χ^2^ = 10.340, *P* = .016, Cramér *V* = 0.273), with a notably higher proportion of amblyopia patients having at least one parent with myopia of 600 degrees or more.

**Table 1:** Demographic information of participants.

Indicator	Overall (n = 139)	M-H M with A (n = 9)	M-H M without A (n = 64)	Mild M with A (n = 8)	Mild myopia without A (n = 58)	Intergroup comparison
Sex						
Male	74	5	32	3	34	χ^2^ = 1.760 *P* = .624 Cramér *V* = 0.113
Female	65	4	32	5	24	
Age	9.64 ± 2.63	10.78 ± 2.05	9.63 ± 2.71	9.63 ± 1.60	9.48 ± 2.75	*F* = 0.625 *P* = .600 η^2^ = 0.014
Height (cm)	142.65 ± 15.27	147.89 ± 12.02	144.16 ± 14.34	137.13 ± 13.94	140.93 ± 16.71	*F* = 1.158 *P* = .328 η^2^ = 0.025
Rhinitis						
Yes	39	2	22	3	12	χ^2^ = 3.330 *P* = .343 Cramér *V* = 0.155
No	100	7	42	5	46	
Parental myopia						
Yes	43	7	18	3	15	χ^2^ = 10.340 *P* = .016 Cramér *V* = 0.273
No	96	2	46	5	43	

A = amblyopia, M =** **myopia, M-H = moderate to high.

### 3.2. Changes in visual acuity during the intervention period

The changes in visual acuity during the intervention period are shown in Table 2. Both binocular visual acuity, better eye visual acuity, and poor eye visual acuity progressively improved over the course of the intervention. The greatest improvement was observed in binocular visual acuity (Cohen *d* = 1.462), while the least improvement was observed in better eye visual acuity (Cohen *d* = 1.273). The 8-week intervention achieved a large effect size, with further improvement observed at the 12-week follow-up. Clinically, the mean visual acuity of the better eye improved from 0.488 (equivalent to rows 7–8 on the standard log minimum angle of resolution chart) to 0.686 (rows 9–10), while the poorer eye improved from 0.332 (rows 6–7) to 0.552 (rows 8–9). These changes correspond to an approximate 2-line gain on the acuity chart, with the minimum angle of resolution reduced to approximately 63% of the baseline value.

**Table 2:** Changes in visual acuity during the intervention period in participants.

Indicator	Time (wk)	Overall (n = 139)	Cohen *d* (95% CI)
Both eyes	0	0.549 ± 0.316	–
	4	0.652 ± 0.347*	0.892 (0.694, 1.088)
	8	0.729 ± 0.359*	1.252 (1.028, 1.472)
	12	0.788 ± 0.374*	1.462 (1.222, 1.701)
Better eye	0	0.488 ± 0.310	–
	4	0.560 ± 0.335*	0.646 (0.462, 0.828)
	8	0.621 ± 0.332*	1.157 (0.941, 1.371)
	12	0.686 ± 0.373*	1.273 (1.048, 1.496)
Worse eye	0	0.332 ± 0.226	–
	4	0.417 ± 0.258*	0.914 (0.715, 1.111)
	8	0.491 ± 0.276*	1.402 (1.166, 1.635)
	12	0.552 ± 0.320*	1.346 (1.115, 1.574)

CI = confidence interval.

* Indicates a statistically significant difference from baseline (0 wk), with *P*-values < .001 based on paired *t*-tests. Cohen *d* effect sizes represent the magnitude of change compared to the baseline (0 wk).

### 3.3. Visual acuity changes among participants with different degrees of myopia and amblyopia

**Figure 3.**
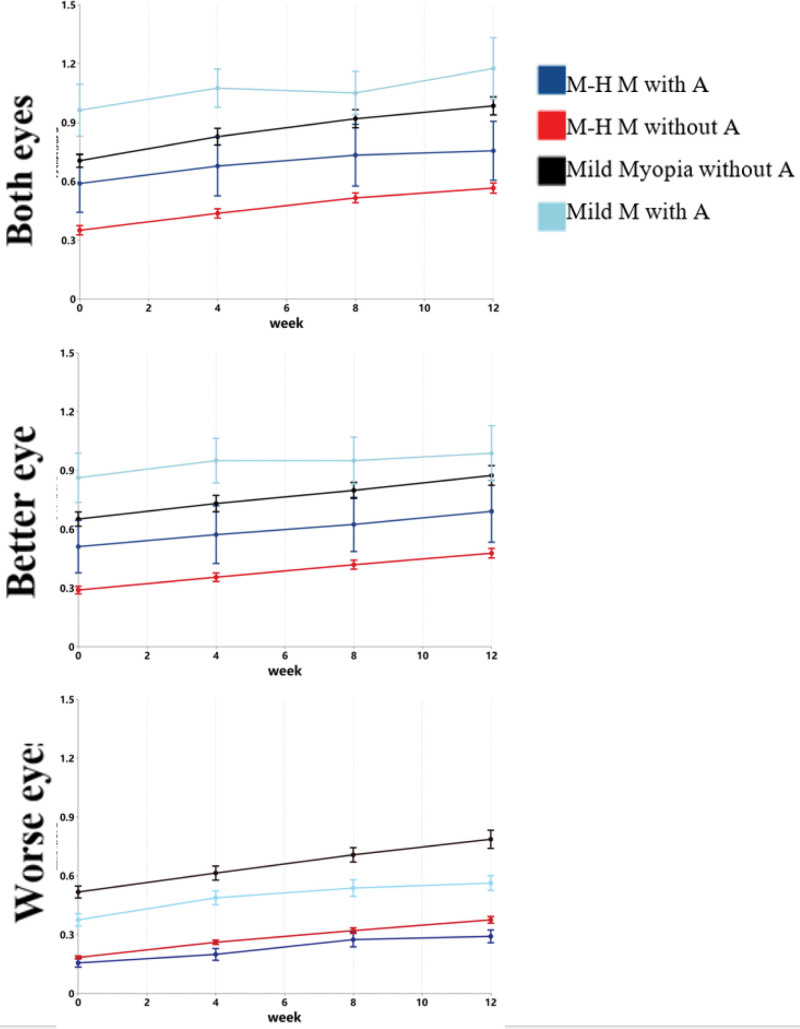
Changes in visual acuity in participants with different degrees of myopia and amblyopia.

As shown in Table 3 and Figure 3, there were considerable differences in visual acuity among participants with varying degrees of myopia and amblyopia, particularly in the worse eye. The extent of improvement following the intervention also varied across groups, making direct between-group comparisons inappropriate. These findings suggest that both the severity of myopia and the presence of amblyopia are important influencing factors and should be included in subsequent analyses as key variables. Generally speaking, the improvement in visual acuity among amblyopia patients is relatively small.

**Table 3:** Changes in visual acuity in participants with different degrees of myopia and amblyopia.

Indicator	Time (wk)	M		A		M-H M with A (n = 9)	M-H M without A (n = 64)	Mild M with A (n = 8)	Mild myopia without A (n = 58)	Intergroup comparison	
		Mild (n = 66)	M-H (n = 73)	Yes (n = 17)	No (n = 122)					*F*	*P*
Both eyes	0	0.736 ± 0.281	0.380 ± 0.243	0.765 ± 0.442	0.519 ± 0.284	0.589 ± 0.440	0.351 ± 0.189	0.963 ± 0.374	0.705 ± 0.254	28.284	<.001
	4	0.858 ± 0.327	0.466 ± 0.246	0.865 ± 0.423	0.623 ± 0.326	0.678 ± 0.456	0.437 ± 0.188	1.075 ± 0.276	0.828 ± 0.324	26.733	<.001
	8	0.935 ± 0.346	0.543 ± 0.254	0.882 ± 0.425	0.707 ± 0.345	0.733 ± 0.472	0.516 ± 0.199	1.050 ± 0.312	0.919 ± 0.350	21.999	<.001
	12	1.008 ± 0.364	0.589 ± 0.254	0.953 ± 0.484	0.765 ± 0.353	0.756 ± 0.450	0.566 ± 0.209	1.175 ± 0.443	0.985 ± 0.350	23.367	<.001
Better eye	0	0.677 ± 0.293	0.317 ± 0.210	0.677 ± 0.410	0.462 ± 0.286	0.511 ± 0.401	0.289 ± 0.154	0.863 ± 0.354	0.652 ± 0.277	29.088	<.001
	4	0.758 ± 0.323	0.382 ± 0.230	0.750 ± 0.424	0.534 ± 0.314	0.572 ± 0.441	0.355 ± 0.173	0.950 ± 0.321	0.731 ± 0.317	25.453	<.001
	8	0.817 ± 0.318	0.444 ± 0.231	0.778 ± 0.405	0.599 ± 0.317	0.624 ± 0.415	0.419 ± 0.184	0.950 ± 0.338	0.798 ± 0.314	24.067	<.001
	12	0.888 ± 0.382	0.504 ± 0.251	0.831 ± 0.452	0.666 ± 0.358	0.691 ± 0.472	0.477 ± 0.195	0.988 ± 0.398	0.874 ± 0.381	18.440	<.001
Worse eye	0	0.550 ± 0.223	0.180 ± 0.063	0.259 ± 0.135	0.342 ± 0.235	0.156 ± 0.063	0.183 ± 0.063	0.375 ± 0.089	0.517 ± 0.231	49.311	<.001
	4	0.599 ± 0.261	0.253 ± 0.091	0.335 ± 0.174	0.429 ± 0.266	0.199 ± 0.090	0.261 ± 0.090	0.488 ± 0.099	0.614 ± 0.273	39.391	<.001
	8	0.686 ± 0.272	0.315 ± 0.115	0.398 ± 0.175	0.504 ± 0.286	0.274 ± 0.111	0.320 ± 0.115	0.538 ± 0.119	0.707 ± 0.281	40.795	<.001
	12	0.759 ± 0.339	0.365 ± 0.133	0.419 ± 0.171	0.571 ± 0.332	0.291 ± 0.098	0.375 ± 0.134	0.563 ± 0.106	0.786 ± 0.352	31.261	<.001

A = amblyopia, M =** **myopia, M-H = moderate to high.

### 3.4. Changes in visual acuity in participants with myopia and amblyopia under different family intervention measures

**Figure 4.**
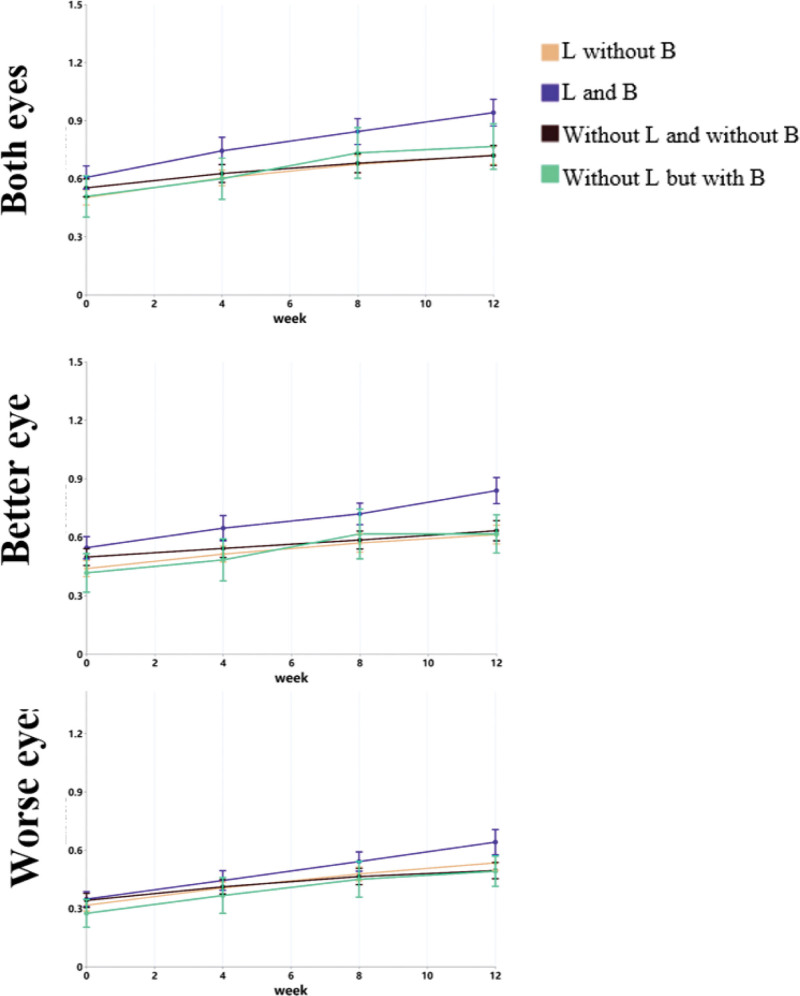
Changes in visual acuity in participants with myopia and amblyopia under different family intervention measures.

As shown in Table 4 and Figure 4, participants who took lutein and wore blue light-blocking glasses exhibited the greatest improvement in visual acuity, while those who did not take lutein and did not wear blue light-blocking glasses showed the least improvement.

**Table 4:** Changes in visual acuity in participants with myopia and amblyopia under different family intervention measures.

Indicator	Time (wk)	L		B		L and B (n = 41)	L without B (n = 49)	Without L but with B (n = 6)	Without L and without B (n = 43)	Intergroup comparison	
		Yes (n = 90)	No (n = 49)	Yes (n = 47)	No (n = 92)					*F*	*P*
Both eyes	0	0.550 ± 0.329	0.547 ± 0.295	0.594 ± 0.365	0.526 ± 0.287	0.607 ± 0.379	0.503 ± 0.275	0.508 ± 0.262	0.553 ± 0.301	0.832	.479
	4	0.668 ± 0.373	0.624 ± 0.295	0.726 ± 0.431	0.615 ± 0.290	0.744 ± 0.450	0.604 ± 0.282	0.600 ± 0.261	0.627 ± 0.302	1.403	.245
	8	0.752 ± 0.379	0.687 ± 0.318	0.830 ± 0.414	0.677 ± 0.317	0.844 ± 0.428	0.675 ± 0.316	0.733 ± 0.320	0.680 ± 0.321	2.093	.104
	12	0.822 ± 0.394	0.726 ± 0.331	0.919 ± 0.426	0.721 ± 0.328	0.942 ± 0.441	0.721 ± 0.320	0.767 ± 0.288	0.720 ± 0.339	3.472	.018
Better eye	0	0.488 ± 0.327	0.488 ± 0.278	0.530 ± 0.352	0.467 ± 0.286	0.546 ± 0.365	0.439 ± 0.288	0.417 ± 0.242	0.498 ± 0.284	1.010	.390
	4	0.574 ± 0.355	0.535 ± 0.297	0.626 ± 0.399	0.527 ± 0.294	0.646 ± 0.414	0.513 ± 0.288	0.483 ± 0.264	0.542 ± 0.304	1.379	.252
	8	0.638 ± 0.349	0.589 ± 0.299	0.706 ± 0.348	0.577 ± 0.317	0.720 ± 0.355	0.570 ± 0.332	0.617 ± 0.313	0.585 ± 0.302	1.776	.155
	12	0.716 ± 0.394	0.631 ± 0.325	0.811 ± 0.413	0.623 ± 0.335	0.839 ± 0.427	0.613 ± 0.335	0.617 ± 0.240	0.633 ± 0.338	3.464	.018
Worse eye	0	0.331 ± 0.236	0.334 ± 0.230	0.339 ± 0.238	0.328 ± 0.222	0.348 ± 0.246	0.317 ± 0.209	0.275 ± 0.175	0.342 ± 0.237	0.295	.829
	4	0.423 ± 0.269	0.406 ± 0.238	0.444 ± 0.323	0.406 ± 0.215	0.444 ± 0.323	0.406 ± 0.215	0.367 ± 0.225	0.411 ± 0.242	0.259	.855
	8	0.507 ± 0.282	0.463 ± 0.266	0.530 ± 0.307	0.471 ± 0.259	0.542 ± 0.318	0.478 ± 0.248	0.450 ± 0.226	0.464 ± 0.273	0.667	.574
	12	0.583 ± 0.345	0.495 ± 0.263	0.622 ± 0.393	0.516 ± 0.271	0.642 ± 0.413	0.535 ± 0.270	0.492 ± 0.191	0.495 ± 0.273	1.664	.178

A = amblyopia, B = blue light-blocking glasses, L =** **lutein supplementation.

### 3.5. Analysis of factors influencing the effectiveness of the vision intervention

The results of repeated measures ANOVA for binocular vision are presented in Table 5. A significant main effect of time was observed (*F*_(3, 135)_ = 57.423, *P* < .001, partial η^2^ = 0.313). Additionally, there were significant interaction effects between time and amblyopia status (*F*_(3, 135)_ = 2.808, *P* = .039, partial η^2^ = 0.022), and between time and blue light protection (*F*_(3, 135)_ = 3.681, *P* = .021, partial η^2^ = 0.028). All other interaction effects were not statistically significant.

**Table 5:** Repeated measures ANOVA of factors affecting changes in visual acuity.

Dependent variable	Effect	*F*	*P*	Partial η^2^
Both eyes	Time	57.423	<.001	0.313
	Time × Degree of myopia	0.619	.563	0.005
	Time × Amblyopia status	2.808	.039	0.022
	Time × Lutein	0.739	.498	0.006
	Time × Blue light-blocking glasses	3.681	.021	0.028
	Time × Degree of myopia × Amblyopia status	1.422	.241	0.011
	Time × Degree of myopia × Lutein	1.046	.366	0.008
	Time × Degree of myopia × Blue light-blocking glasses	0.166	.877	0.001
	Time × Amblyopia status × Lutein	0.103	.927	0.001
	Time × Amblyopia status × Blue light-blocking glasses	0.410	.695	0.003
	Time * Lutein * Blue light-blocking glasses	0.403	.700	0.003
Better eye	Time	39.522	<.001	0.239
	Time × Degree of myopia	1.543	.203	0.012
	Time × Amblyopia status	1.889	.140	0.015
	Time × Lutein	1.310	.272	0.010
	Time × Blue light-blocking glasses	2.479	.070	0.019
	Time × Degree of myopia × Amblyopia status	1.830	.150	0.014
	Time × Degree of myopia * Lutein	0.882	.438	0.007
	Time × Degree of myopia × Blue light-blocking glasses	0.987	.390	0.008
	Time × Amblyopia status × Lutein	0.517	.644	0.004
	Time × Amblyopia status × Blue light-blocking glasses	0.390	.731	0.003
	Time × Lutein × Blue light-blocking glasses	2.214	.096	0.017
Worse eye	Time	59.384	<.001	0.320
	Time × Degree of myopia	1.488	.228	0.012
	Time × Amblyopia status	2.624	.074	0.020
	Time × Lutein	1.383	.253	0.011
	Time × Blue light-blocking glasses	0.971	.379	0.008
	Time × Degree of myopia × Amblyopia status	0.452	.635	0.004
	Time × Degree of myopia × Lutein	0.634	.530	0.005
	Time × Degree of myopia × Blue light-blocking glasses	1.053	.350	0.008
	Time × Amblyopia status × Lutein	0.086	.932	0.001
	Time × Amblyopia status × Blue light-blocking glasses	0.265	.765	0.002
	Time × Lutein × Blue light-blocking glasses	0.501	.604	0.004

ANOVA = analysis of variance.

For the better-seeing eye, the main effect of time was also significant (*F*_(3, 135)_ = 39.522, *P* < .001, partial η^2^ = 0.239), but no significant interaction effects were found. For the worse-seeing eye, the main effect of time was significant (*F*_(3, 135)_ = 59.384, *P* < .001, partial η^2^ = 0.320), while all interaction effects remained nonsignificant.

**Figure 5.**
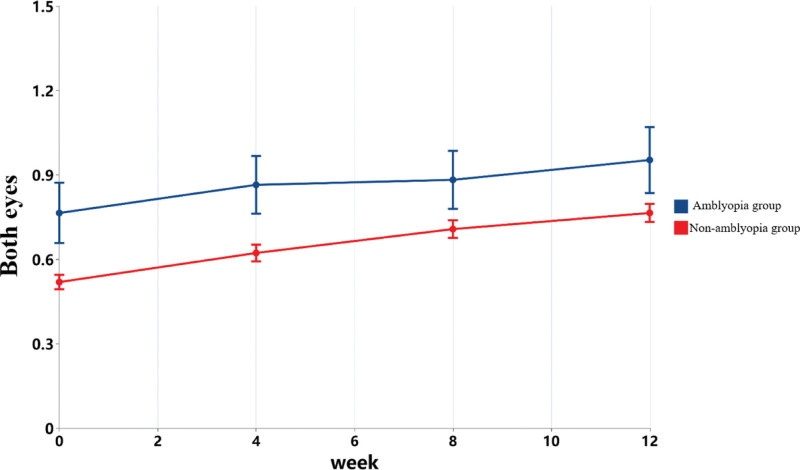
Interaction effect of time and amblyopia status.

Further simple effect analyses were conducted on factors with significant interaction effects. As shown in Figure 5, the binocular visual acuity of amblyopia patients was consistently better than that of non-amblyopia patients. At 0 weeks, the mean difference in binocular visual acuity was 0.207 (95% CI: 0.071–0.343, *P* = .003); at 4 weeks, it was 0.206 (95% CI: 0.057–0.356, *P* = .007); at 8 weeks, it was 0.129 (95% CI: −0.029–0.288, *P* = .109); and at 12 weeks, it was 0.141 (95% CI: −0.021–0.302, *P* = .087). As the intervention progressed, the differences in both binocular visual acuity and the visual acuity of the worse eye between amblyopia patients and non - amblyopia patients gradually narrowed. The difference between the two groups was no longer significant at the 8th week.

**Figure 6.**
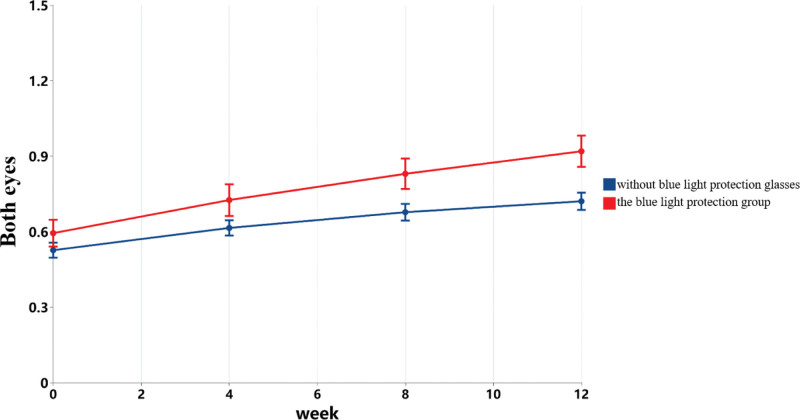
Interaction effect of time and blue light-blocking glasses usage.

As illustrated in Figure 6, participants using blue light protection consistently outperformed those without it in terms of binocular vision. At week 0, the mean difference was 0.135 (95% CI: 0.012–0.258, *P* = .031); at week 4, 0.160 (95% CI: 0.024–0.295, *P* = .021); at week 8, 0.230 (95% CI: 0.086–0.374, *P* = .002); and at week 12, 0.255 (95% CI: 0.109–0.402, *P* < .001). This suggests that the vision improvement effect was more pronounced in the blue light protection group, with the between-group difference widening over time.

### 3.6. Safety evaluation

A total of 139 patients were included in this study. After excluding the cases of termination, none of the patients who participated in the intervention showed obvious skin damage, pain, allergic reactions, or other adverse reactions and events.

## 4. Discussion

This study aimed to investigate the effects of Traditional Chinese Medicine Tuina massage on visual changes in myopic children and adolescents under various intervention strategies and to assess the impact of different home-based preventive factors on vision improvement.

The results revealed that after 12 weeks of intervention, significant improvements were observed in visual acuity for both eyes, as well as in the better and worse eyes, with the degree of improvement progressively increasing over time.

The vision conditioning program proposed in this study comprises several key components: acupressure, flexible spinal manipulation, vision exercises, physical activity, and home-based preventive measures. These components collectively contribute to significant improvements in vision. The core element of the intervention is Traditional Chinese Medicine Tuina massage, which refines the application of techniques such as combining acupressure with meridian massage to enhance the function of the 5 internal organs, particularly the spleen, stomach, liver, and kidneys, thereby improving vision. This approach is consistent with previous studies,^[24–26]^ demonstrating effects similar to those of acupuncture while avoiding the pain and risks associated with acupuncture.

Meridian-based acupressure is used to regulate the internal organs, with a specific focus on the spleen, stomach, liver, gallbladder, and kidneys. According to traditional Chinese medicine, “The qi of the 5 internal organs ascends to the eyes, and the eyes receive blood to support vision.” Dysfunction in the internal organs is considered the primary cause of vision problems.^[10]^ Data from Zhejiang Medical University indicate that 66.9% of liver disease patients also suffer from ocular issues, including vision decline and other related complications.^[27]^ Moreover, dysregulation of the spleen and stomach significantly increases the risk of ocular diseases.^[28]^

This study protocol integrates and builds upon prior research and emphasizes body correction techniques, such as regulating the bladder meridian along the back, correcting spinal scoliosis, and adjusting the suboccipital muscle group. The central techniques involve stimulating reflex zones along the bladder meridian on the back, corresponding to the internal organs. Spinal scoliosis, pelvic tilt, and other morphological changes can lead to compression of muscles, nerves, and blood vessels, which in turn compresses the internal organs, obstructs meridians, and hinders the normal circulation of qi and blood to the head and face. Adjusting the spinal position ensures the normal generation and transmission of qi and blood in the internal organs.

The suboccipital muscle group contains arteries and nerves. The techniques focus on alleviating stiffness or spasms in the suboccipital muscles, ensuring that the vertebral arteries and nerves are not compressed, thus facilitating better circulation of qi and blood to the head and face and ensuring adequate blood supply to the eyes.

The results of this study suggest that the degree of myopia and the presence of amblyopia significantly influence the extent of vision improvement. Among the subjects with moderate to severe myopia accompanied by amblyopia, the degree of visual acuity improvement after the intervention was smaller compared to other groups, especially in the change of visual acuity in the worse - seeing eye. On the contrary, patients with mild myopia without amblyopia showed the greatest improvement. These findings further support the importance of individualized intervention programs. Among these factors, whether a patient has amblyopia is of greater importance. There was a significant interaction effect between time and the presence of amblyopia. As time passed, the visual acuity improvement of patients with myopia and amblyopia was significantly less than that of myopic patients without amblyopia. Previous studies have shown that children with higher degrees of myopia typically exhibit greater improvements in vision correction treatments, while amblyopic patients tend to show slower progress, particularly when high myopia is also present. Although visual training can accelerate the recovery of amblyopic patients, their recovery is generally more challenging compared to patients with myopia alone.^[29,30]^

Regarding home-based preventive measures, participants who took lutein supplements and wore blue light-blocking glasses exhibited more significant improvements in vision. Notably, by the 12th week, the group using both lutein and blue light-blocking glasses demonstrated the greatest improvement in visual acuity, while those who did not adopt these measures experienced slower improvement. The interaction between wearing blue light-blocking glasses and time was significant, suggesting that limiting screen time and using blue light-blocking glasses are critical preventive measures. The blue light emitted by screens and intense light stimulation causes the pupils to constrict and dilate excessively, affecting the normal functioning of the eyes and ocular structures. Blue light-blocking glasses may help alleviate eye fatigue, especially during prolonged electronic device use, though the specific benefits remain inconclusive.^[31]^ Traditional Chinese medicine holds that vision is primarily associated with the spleen, and yellow-colored foods benefit the spleen.^[32]^ Consuming lutein may therefore have a positive effect on the spleen. Studies have shown that continuous lutein supplementation for 16 weeks increases macular pigment optical density, improves the clarity of visible objects, and significantly reduces visual function decline due to glare.^[33]^ Supplementing 12 mg of lutein daily was found to be more effective than 6 mg per day in young adults aged 22 to 30 years,^[34]^ suggesting that higher doses may be more effective. In this study, although improvements were observed in the group that took lutein supplements and wore blue-light blocking glasses simultaneously, the benefits of taking lutein supplements alone (as an independent factor) for visual acuity did not reach statistical significance. This could be due to the need for a higher dosage or a larger sample size. High-dose lutein supplementation generally does not cause adverse reactions,^[35]^ but further research is needed to confirm its safety for myopic children and adolescents.

## 5. Limitations of the study

This study adopted a single–group experimental design. Due to the absence of a control group, it was difficult to precisely determine the effect size. Moreover, the study was susceptible to interferences such as natural changes, practice effects, and selective biases, which led to relatively low internal validity. The intervention in this study integrated physical exercise, Traditional Chinese Medicine Tuina massage, and home - based preventive measures. In particular, it was impossible to clearly distinguish the benefits of the first two components. Limited by the statistical power associated with the sample size, subgroup analyses based on the age and gender of the subjects could not be carried out. The home-based intervention measures mainly focused on anti-blue-light glasses and lutein supplements. However, it was challenging for the researchers to control certain home - based intervention measures, such as diet adjustment and outdoor physical activities.

## Acknowledgments

We are grateful to all participants who contributed to this study.

## Author contributions

**Data analysis:** Jun Wu, Jihan Huang.

**Data collection:** Peng Wang, Feng Ding.

**Writing – editing:** Shuqi Jia.

**Data curation:** Caiyun Wang.

**Methodology:** Qianmin Su.

**Writing – original draft:** Jun Wu, Peng Wang.

**Writing – review and editing:** Jing Wang.

## References

[1] Cao H Cao X Cao Z Zhang L Han Y Guo C. The prevalence and causes of pediatric uncorrected refractive error: pooled data from population studies for global burden of disease (GBD) sub-regions. PLoS One. 2022;17:e0268800. 35776717 10.1371/journal.pone.0268800 PMC9249246

[2] Holden BA Fricke TR Wilson DA. Global prevalence of myopia and high myopia and temporal trends from 2000 through 2050. Ophthalmology. 2016;123:1036–42. 26875007 10.1016/j.ophtha.2016.01.006

[3] Pan CW Wu RK Liu H Li J Zhong H. Types of lamp for homework and myopia among Chinese school-aged children. Ophthalmic Epidemiol. 2018;25:250–6. 29281362 10.1080/09286586.2017.1420204

[4] Jiang J Kong K Lin F; Glaucoma Suspects with High Myopia Study Group. Longitudinal changes of retinal nerve fiber layer and ganglion cell-inner plexiform layer in highly myopic glaucoma: a 3-year cohort study. Ophthalmology. 2025;132:644–53. 39842730 10.1016/j.ophtha.2025.01.014

[5] Micelli-Ferrari T Vendemiale G Grattagliano I. Role of lipid peroxidation in the pathogenesis of myopic and senile cataract. Br J Ophthalmol. 1996;80:840–3. 8942384 10.1136/bjo.80.9.840 PMC505624

[6] Benahmed R Dormegny L Gaudric A. Perivascular chorioretinal atrophy: an unusual feature in pathologic myopia eyes. Am J Ophthalmol. 2025;271:498–506. 39746594 10.1016/j.ajo.2024.12.022

[7] Abdelmassih Y Lecoge R El Hassani M. Risk factors for retinal detachment in Marfan syndrome after pediatric lens removal. Am J Ophthalmol. 2024;266:190–5. 38821454 10.1016/j.ajo.2024.05.003

[8] Feng Y Tingting Li Zhenshan G. Distribution of poor vision and screened myopia among children and adolescents in six provinces of China. Chin J Health Educ. 2024;40:483–486 + 498.

[9] Gao RY Gao JR Zhao HY. Mechanism of tonifying Qi by traditional Chinese medicine from mitochondrial dynamics. China J Chin Materia Med. 2023;48:3684–92. in Chinese. 10.19540/j.cnki.cjcmm.20230417.601 37475000

[10] Xin Y Zefeng K Hongrui S. Discussion on the pathogenesis and treatment of myopia based on the “Theory of Jingjin Imbalance” and biorhythm. J Beijing Univ Chin Med. 2023;46:1750–5.

[11] Rui Z Dun’e S Cuizhu W. Treatment of myopia in adolescents by “Adjusting Spine and Raising Yang” from the theory of internal organs and meridians. China J Chin Ophthalmol. 2025;35:153–7.

[12] Zhenyu W Chun Z Jun Z Shuchun S. Overview of research on cervical visual impairment. J Tradit Chin Orthop Traumatol. 2005;8:74–75.

[13] Yvon C Adams A Mclauchlan D Ramsden C. Headache and transient visual loss as the only presenting symptoms of vertebral artery dissection: a case report. J Med Case Rep. 2016;10:105. 27113722 10.1186/s13256-016-0893-8 PMC4843209

[14] Kashif M Manzoor N Safdar R Khan H Farooq M Wassi A. Effectiveness of sustained natural apophyseal glides in females with cervicogenic headache: a randomized controlled trial. J Back Musculoskelet Rehabil. 2022;35:597–603. 34542060 10.3233/BMR-210018

[15] Grinenko EA Kul’chikov AE Musin RS Morozov SG. The effect of the instability of cervical spine on the hemodynamics in the vertebrobasilar system. Zh Nevrol Psikhiatr Im S S Korsakova. 2014;114:69–75. 24730044

[16] Jianzhong Li Hong Z Wenhai F. Observation on the efficacy of acupuncture - based treatment for 50 cases of myopia in children. Chin Acupunct Moxibustion. 2000;20(S1):213–4.

[17] Li X Zhang H Zhang T. Clinical observation of Zheng’s stunt needling technique in the treatment of juvenile myopia. Chin Acupunct Moxibustion. 2018;38:147–50. 10.13703/j.0255-2930.2018.02.010 29473357

[18] Guo C Huang D Ren H. Study on effect of sticking and pressing ear acupoint combined with local acupoint massage on light and moderate myopia in teenagers. Liaoning J Tradit Chin Med. 2018;45:1962–5.

[19] Wenjun Z. Observation on the efficacy of tuina in the treatment of 60 cases of myopia in children. J Gansu Univ Chin Med. 1998;4:38–39.

[20] Zhou P Zhang YM Hong X. Clinical observation in therapy of orthopaedic-spinal massage combined with local face massage for teenagers pseudomyopia. J Yunnan Univ Chin Med. 2014;37:47–50.

[21] Yang Liang LI Ruiqing L Zhu D. Observation on the efficacy of acupoint pressing in the treatment of 30 cases of children’s pseudomyopia. Chin J Tissue Eng Res. 2001;11:119.

[22] National Administration of Traditional Chinese Medicine. Diagnostic criteria, syndrome classification, and efficacy evaluation of myopia – standards for diagnosis and efficacy evaluation of diseases and syndromes in Traditional Chinese Medicine internal medicine, the industry standard of Traditional Chinese Medicine of the People’s Republic of China (ZY/T001.1-94). J Liaoning Univ Tradit Chin Med. 2019;21:58.

[23] Ophthalmology Branch of Chinese Medical Association (Strabismus and Pediatric Ophthalmology Group). Expert consensus on the prevention and treatment of amblyopia in Chinese children (2021). Chin J Ophthalmol. 2021;57:336–40. 10.3760/cma.j.cn112142-20210109-00014 33915635

[24] Yunqiu C. Observation on the efficacy of comprehensive Traditional Chinese Medicine therapy in the treatment of myopia in adolescents. J Sichuan Tradit Chin Med. 2009;27:110–1.

[25] Changchao F Jingyao L Chen Li. Research progress on the prevention and treatment of myopia in children and adolescents with Tuina as the main method in the past five years. Hubei J Tradit Chin Med. 2025;47:63–6.

[26] Wang T Chen N Zeng X. Prevention and control of myopia in children and adolescents by Chinese medical therapies: a network meta-analysis. World Chin Med. 2024;19:2119–27.

[27] Junqiu C. Ocular clinical manifestations of liver diseases. Chin J Clin Res. 1991;2:60–1.

[28] Mingmei G. Clinical Investigation and Analysis of 100 Dry Eye Patients. Master’s thesis, Shandong University of Traditional Chinese Medicine. 2014. [in Chinese]

[29] Yao Y He Y Wen Y. Factual evidence on digital therapeutics in pediatric amblyopia: insights into rapid axial elongation risk. Ophthalmology. 2025;132:661–70. 39814322 10.1016/j.ophtha.2025.01.005

[30] Jain IS Pillai P Gangwar DN Gopal L Dhir SP. Congenital cataract: management and results. J Pediatr Ophthalmol Strabismus. 1983;20:243–6. 6644486 10.3928/0191-3913-19831101-07

[31] Singh S Keller PR Busija L. Blue-light filtering spectacle lenses for visual performance, sleep, and macular health in adults. Cochrane Database Syst Rev. 2023;8:CD013244. 37593770 10.1002/14651858.CD013244.pub2 PMC10436683

[32] Jinrong H Baoxia Z Meili L. Research on the Spleen and Stomach Theory in Huangdi Neijing. 1st ed. Sunshine Press. [in Chinese]

[33] Machida N Kosehira M Kitaichi N. Clinical effects of dietary supplementation of lutein with high bio-accessibility on macular pigment optical density and contrast sensitivity: a randomized double-blind placebo-controlled parallel-group comparison trial. Nutrients. 2020;12:2966. 32998324 10.3390/nu12102966 PMC7600844

[34] Ma L Lin X-M Zou Z-Y Xu X-R Li Y Xu R. A 12-week lutein supplementation improves visual function in Chinese people with long-term computer display light exposure. Br J Nutr. 2009;102:186–90. 19586568 10.1017/S0007114508163000

[35] Lopresti AL Smith SJ. The effects of lutein/ zeaxanthin (Lute-gen(®)) on eye health, eye strain, sleep quality, and attention in high electronic screen users: a randomized, double-blind, placebo-controlled study. Front Nutr. 2025;12:1522302. 39963662 10.3389/fnut.2025.1522302 PMC11830589

